# Supplementary material for: The causal relationship between the human gut microbiota and pyogenic arthritis: a Mendelian randomization study
Source: Front Cell Infect Microbiol. 2024 Nov 26;14:1452480. doi: 10.3389/fcimb.2024.1452480 (PMC11629706; doi:10.3389/fcimb.2024.1452480)
Supplement: Supplementary file 1 [file DataSheet1.zip › mendelian test/ebi-a-GCST90027746.csv_forest.pdf]

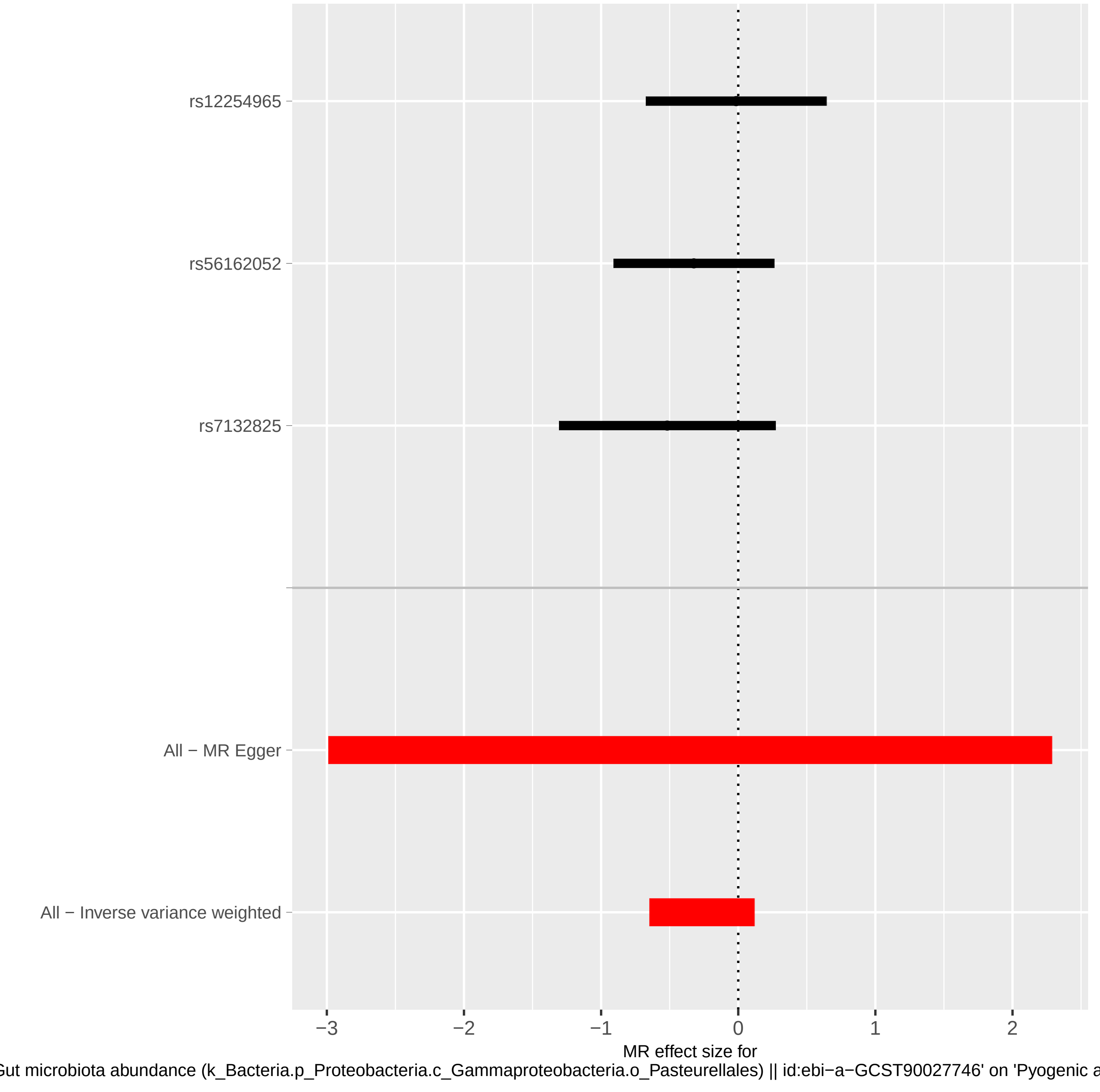

rs12254965
rs56162052
rs7132825
All − MR Egger
All − Inverse variance weighted
−3
−2
−1
0
1
2
MR effect size for
Gut microbiota abundance (k_Bacteria.p_Proteobacteria.c_Gammaproteobacteria.o_Pasteurellales) || id:ebi−a−GCST90027746' on 'Pyogenic a
